# Deletion of UCP1 in Tg2576 Mice Increases Body Temperature and Exacerbates Alzheimer’s Disease-Related Pathologies

**DOI:** 10.3390/ijms24032741

**Published:** 2023-02-01

**Authors:** Cha-Gyun Jung, Hitoshi Yamashita, Reiko Kato, Chunyu Zhou, Hiroaki Matsushita, Tamaki Takeuchi, Mona Abdelhamid, Yuxin Chen, Makoto Michikawa

**Affiliations:** 1 Department of Biochemistry, Graduate School of Medical Sciences, Nagoya City University, 1 Kawasumi, Mizuho-cho, Mizuho-ku, Nagoya 467-8601, Japan; 2 Department of Biomedical Sciences, College of Life and Health Sciences, Chubu University, 1200 Matsumoto-cho, Kasugai 487-8501, Japan

**Keywords:** Alzheimer’s disease, uncoupling protein 1, high body temperature, Aβ generation, BACE1, NEP, tau, heat shock proteins, glial activation, synaptic proteins

## Abstract

We previously demonstrated that the Alzheimer’s disease (AD)-like model mice, Tg2576, housed at a high ambient temperature of 30 °C for 13 months, exhibited increased body temperature, which increased amyloid-β (Aβ) levels and tau stability, leading to tau phosphorylation and ultimately inducing memory impairment. Here, we aimed to exclude the possible effect of environmental factors associated with the difference in ambient temperature (23 °C vs. 30 °C) and to further clarify the effects of elevated body temperature on AD-like pathologies. We generated uncoupling protein 1 (UCP1) deletion in Tg2576 mice, Tg2576/UCP1^−/−^, because UCP1 deletion mice show a sustained rise in body temperature at normal room temperature. As expected, the body temperature in Tg2576/UCP1^−/−^ mice was higher than that in Tg2576/ UCP1^+/+^ mice at 23 °C, which was accompanied by upregulated Aβ levels due to increased β-secretase (BACE1) and decreased neprilysin (NEP) protein levels in the brains of Tg2576/UCP1^−/−^ mice compared with those in the Tg2576/ UCP1^+/+^ mice. Elevated body temperature also increased total tau levels, leading to enhanced phosphorylation, heat shock protein induction, and activated tau kinases. Furthermore, elevated body temperature enhanced glial activation and decreased synaptic protein levels in the brain. Taken together, these findings demonstrate that elevated body temperatures exacerbate AD-like pathologies.

## 1. Introduction

Alzheimer’s disease (AD) is a major chronic neurodegenerative disorder, leading to irreversible cognitive and learning deficits. Aging is thought to be the leading risk factor for AD, which is characterized by the presence of intraneuronal neurofibrillary tangles (NFTs), extracellular senile plaques, and neuroinflammation, which causes neuronal cell death, leading to decline in cognitive function and memory impairment [1]. Amyloid-β (Aβ) is generated by the sequential enzymatic cleavage of amyloid precursor protein (APP) by β- and γ-secretases, leading to Aβ plaque formation [2]. Abnormally modified or misfolded tau proteins are the main pathological features that induce neurodegeneration in AD. The misfolding of tau is the result of either aberrant posttranslational modifications of the tau protein or mutations in its gene [3], resulting mainly from truncation [4] and/or abnormal phosphorylation [5]. In addition, patients with AD suffer from other symptoms that affect their behavior, such as disturbed sleep and circadian rhythms, depression, and anxiety [6,7,8,9]. These behavioral changes might not occur as a result of AD pathology and unfortunately may affect their quality of life.

Since aging is the principal risk factor for AD, understanding the pathological changes caused by aging would disclose new insights into the pathogenesis of AD. Among these, thermoregulatory deficits are thought to be one of the major changes in AD. Indeed, the core temperature is regulated by younger adults better than elderly people [10], with several elderly people displaying a lower body temperature during the day [11], which is thought to be a major risk factor for AD [12]. Interestingly, deficits in optimal thermoregulation roughly coincide with an increased incidence of AD in this population. Although the molecular mechanisms by which lower body temperature induces cognitive dysfunction and exacerbates AD-related pathologies have not yet been fully elucidated, animal and human studies have demonstrated that tau hyperphosphorylation is enhanced by low body temperature [13,14]. Additionally, recent evidence demonstrates that non-cognitive changes in patients with AD include disruptions in the core body temperature and circadian rhythms of locomotor activity, usually characterized by increased nocturnal activity and elevated body temperature [15,16,17,18,19]. Since increased body temperature is associated with impaired cognitive function and AD-like pathology, a higher body temperature might damage the brains of patients with AD [17,18,19].

Brown adipose tissue plays a critical role in adaptive thermogenesis, such as the maintenance of body temperature in a cold environment through the mitochondrial uncoupling protein 1 (UCP1) in mammals, which generates heat by uncoupling oxidative phosphorylation [20,21,22]. UCP1-deficient (UCP1^−/−^) mice cannot maintain body temperature at 5 °C compared with littermate controls [21]; whereas, these mutant mice show an unexpected rise in body temperature with no difference in physical activity compared with that of wild-type mice at normal room temperature (RT) of 23 °C [23]. We also reported that UCP1^−/−^ mice maintain their homeothermy at normal room temperature by suppression of heat liberation from the body [24], which could result in elevated body temperature in these mice. However, the lifespans were not substantially different between wild-type and UCP1^−/−^ mice under standard diet and normal RT conditions [25].

Altered levels and activation of APP and APP-cleaving enzymes, such as α-secretase (ADAM10), β-secretase (BACE1), and γ-secretase component (PS1) are linked with Aβ generation. On the contrary, ATP-binding cassette A1 (ABCA1) and apolipoprotein E (ApoE) are involved in Aβ clearance, and insulin-degrading enzyme (IDE) and neprilysin (NEP) are involved in Aβ degradation [26,27]. We recently demonstrated that the AD-like model mice, Tg2576, housed at 30 °C for 13 months, showed increased body temperature, which enhanced Aβ generation via increased BACE1 protein levels and decreased NEP protein levels. In addition to Aβ generation, high body temperature also increased the levels of total tau, stress-stimulated kinases such as c-jun N-terminal kinase (JNK), and heat shock proteins (HSPs), resulting in the enhancement of tau phosphorylation at 30 °C, which consequently caused memory impairment [26]. However, in addition to higher body temperatures, some environmental factors associated with differences in ambient temperature (23 °C vs. 30 °C) can also affect memory function and AD-like pathologies. Therefore, to exclude the possible effects of environmental factors and to further clarify the effects of high body temperature on AD-like pathologies, we generated Tg2576/UCP1^−/−^ mice in this study. Here, we report that compared with Tg2576/ UCP1^+/+^ mice, Tg2576/UCP1^−/−^ mice with elevated body temperatures demonstrated exacerbated AD-like pathologies due to increased Aβ, HSPs, phosphorylated and total tau, and tau kinases, as well as enhanced glial activation and decreased synaptic protein levels.

## 2. Results

### 2.1. Effects of UCP1 Deletion in Tg2576 Mice on Core Body Temperature

**Figure 1:**
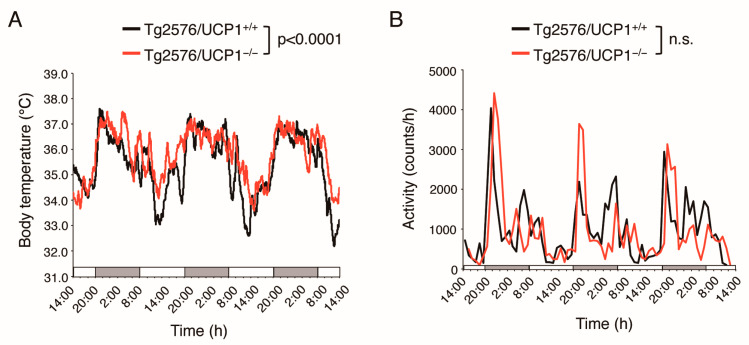
UCP1 deletion in Tg2576 mice increases core body temperature at 23 °C. (**A**) Circadian rhythms of average core body temperature and (**B**) circadian rhythms of physical activity in Tg2576/UCP1^+/+^ and Tg2576/UCP1^−/−^ mice were analyzed using the VitalView system at 15 months of age. The results shown are mean ± SD, and significant differences between two groups were assessed by repeated measures ANOVA. n.s., no significant difference.

We generated Tg2576/UCP1^−/−^ and Tg2576/ UCP1^+/+^ mice to investigate the effects of elevated body temperature on AD-like pathologies in Tg2576 mice. First, we measured core body temperature and physical activity using a non-invasive monitoring system in 15-month-old Tg2576/UCP1^−/−^ and Tg2576/ UCP1^+/+^ mice. The core temperature was significantly higher during the dark and light phases in Tg2576/UCP1^−/−^ mice than in Tg2576/ UCP1^+/+^ (light phase: 34.43 ± 0.33 and 35.21 ± 0.36 °C, *p* < 0.0001; dark phase: 36.34 ± 0.30 and 36.49 ± 0.22 °C, *p* < 0.01, in Tg2576/UCP1^+/+^ and Tg2576/UCP1^−/−^ mice, respectively, Figure 1A). However, there were no significant differences in physical activity between the two groups (light phase: 517 ± 115 and 519 ± 49 counts/h; dark phase: 1479 ± 223 and 1366 ± 465 counts/h, in Tg2576/ UCP1^+/+^ and Tg2576/UCP1^−/−^ mice, respectively, Figure 1B). These results demonstrate sustained high body temperatures in Tg2576/UCP1^−/−^ mice relative to Tg2576/ UCP1^+/+^ mice, consistent with previous findings in UCP1^−/−^ mice [23].

### 2.2. Effect of UCP1 Deletion in Tg2576 Mice on Aβ Generation

**Figure 2:**
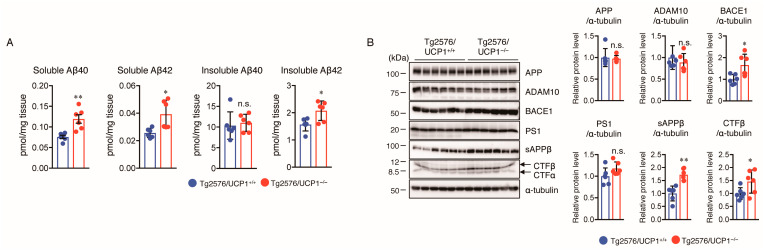
UCP1 deletion in Tg2576 mice increases BACE1 level leading to an increase in Aβ levels in the cortex of brain. (**A**) The levels of soluble and insoluble Aβ40 and Aβ42 in the cortex of brain of 17-month-old mice were measured by sandwich Aβ ELISA. ((**B**), left panel) Representative immunoblots showing the cortical levels of APP, ADAM10, BACE1, PS1, sAPPβ, CTFβ, and α-tubulin in Tg2576/UCP1^+/+^ and Tg2576/UCP1^−/−^ mice. ((**B**), right panels) Relative protein levels quantified by densitometry. The results shown are mean ± SD, *n* = 6 for each group, * *p* < 0.05, ** *p* < 0.01, vs. Tg2576/UCP1^+/+^, n.s., no significant difference, as determined by Student’s *t*-test.

Next, we determined the levels of soluble and insoluble Aβ40 and Aβ42 in the cortex of Tg2576/UCP1^+/+^ and Tg2576/UCP1^−/−^ mice at 17 months of age using Aβ ELISA. The results revealed that soluble Aβ40 and Aβ42 levels were markedly higher in the cortex of Tg2576/UCP1^−/−^ mice than in Tg2576/UCP1^+/+^ mice (Figure 2A). Although there were no significant differences in the level of insoluble Aβ40 between the two groups, the insoluble Aβ42 level was markedly higher in Tg2576/UCP1^−/−^ mice than in Tg2576/UCP1^+/+^ mice (Figure 2A). To investigate the mechanisms by which the high body temperature increased the Aβ generation, we evaluated the protein levels of APP, ADAM10, BACE1, and PS1 in cortical homogenates of mice using Western blotting. We found that Tg2576/UCP1^−/−^ mice had considerably higher protein levels of BACE1 than those in Tg2576/UCP1^+/+^ mice; whereas, there were no significant differences the protein levels of APP, ADAM10, and PS1 between the two groups (Figure 2B). Soluble APPβ (sAPPβ) and the C-terminal fragment β (CTFβ) could be generated by cleaving APP by BACE1. Therefore, we further evaluated the protein levels of sAPPβ, CTFα, and CTFβ using Western blotting. As expected, the levels of sAPPβ and CTFβ were significantly higher in the cortex of Tg2576/UCP1^−/−^ mice than in Tg2576/UCP1^+/+^ mice (Figure 2B). These results are consistent with those from Tg2576 mice housed at 30 °C, and indicate that the high body temperature observed in Tg2576/UCP1^−/−^ mice promotes Aβ generation via the induction of BACE1 expression.

### 2.3. Effect of UCP1 Deletion in Tg2576 Mice on Aβ Degradation and Clearance

**Figure 3:**
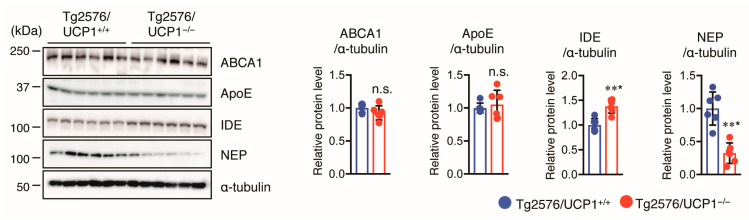
UCP1 deletion in Tg2576 mice increases IDE levels and decreases NEP levels. Representative immunoblots showing the cortical levels of ABCA1, ApoE, IDE, NEP, and α-tubulin in 17-month-old Tg2576/UCP1^+/+^ and Tg2576/UCP1^−/−^ mice (left panel). Relative protein levels quantified by densitometry (right panels). The results shown are mean ± SD, *n* = 6 for each group, *** *p* < 0.001, vs. Tg2576/UCP1^+/+^, n.s., no significant difference, as determined by Student’s *t*-test.

We observed that insoluble Aβ40 levels were not different between Tg2576/UCP1^+/+^ and Tg2576/UCP1^−/−^ mice; whereas, insoluble Aβ42 levels were significantly higher in the cortex of Tg2576/UCP1^−/−^ mice than in Tg2576/UCP1^+/+^ mice. Thus, we hypothesized that the high body temperature observed in Tg2576/UCP1^−/−^ mice may be involved in Aβ accumulation. Therefore, we assessed the levels of ABCA1, ApoE, IDE, and NEP using Western blotting. We found that although the levels of ABCA1 and ApoE were not different between the two groups, NEP levels were significantly decreased, and IDE levels were significantly increased in the cortex of Tg2576/UCP1^−/−^ mice compared with those in Tg2576/UCP1^+/+^ mice (Figure 3). These findings are consistent with those from Tg2576 mice housed at 30 °C, which indicates that high body temperature accelerates Aβ42 accumulation via reduced NEP levels.

### 2.4. Effect of UCP1 Deletion in Tg2576 Mice on Tau Pathology

**Figure 4:**
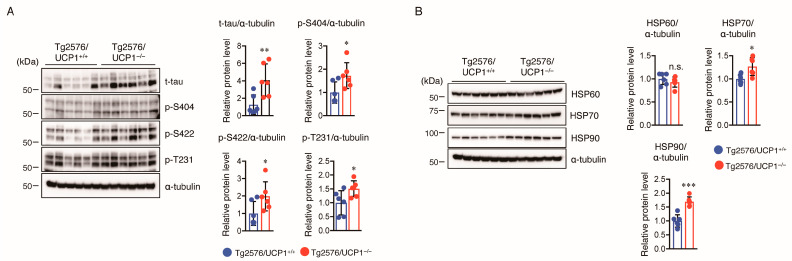
UCP1 deletion in Tg2576 mice increases levels of total tau, phosphorylated tau, and heat shock proteins in the cortex of brain. ((**A**), left panel) Representative immunoblots showing the cortical levels of total- (t-) and phosphorylated- (p-) tau on multiple sites (Ser404, Ser422, and Thr231), and α-tubulin in 17-month-old Tg2576/UCP1^+/+^ and Tg2576/UCP1^−/−^ mice (left panel). ((**A**), right panels) Relative protein levels quantified by densitometry. ((**B**), left panel) Representative immunoblots showing the cortical levels of HSP60, HSP70, HSP90, and α-tubulin in 17-month-old Tg2576/UCP1^+/+^ and Tg2576/UCP1^−/−^ mice. ((**B**), right panels) Relative protein levels quantified by densitometry. The results shown are mean ± SD, *n* = 6 for each group, * *p* < 0.05, ** *p* < 0.01, *** *p* < 0.001 vs. Tg2576/UCP1^+/+^, n.s., no significant difference, as determined by Student’s *t*-test.

**Figure 5:**
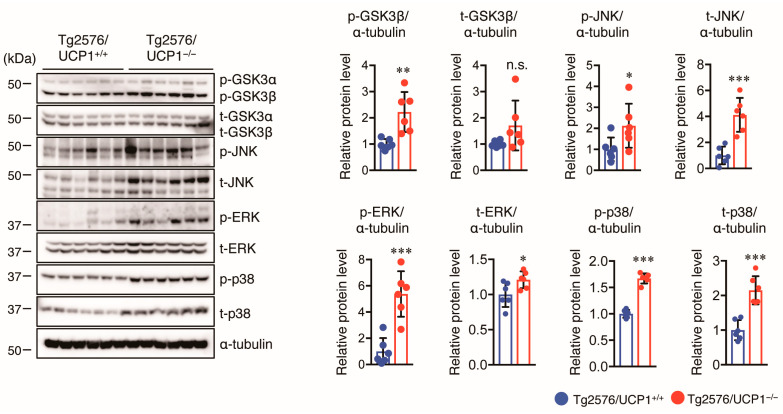
UCP1 deletion in Tg2576 mice enhances the phosphorylation of tau kinases. Representative immunoblots showing the cortical levels of total (t-) and phosphorylated-(p-) GSK3α/β, JNK, ERK, p38, and α-tubulin in 17-month-old Tg2576/UCP1^+/+^ and Tg2576/UCP1^−/−^ mice (left panel). Relative protein levels quantified by densitometry (right panels). The results shown are mean ± SD, *n* = 6 for each group, * *p* < 0.05, ** *p* < 0.01, *** *p* < 0.001 vs. Tg2576/UCP1^+/+^, n.s., no significant difference, as determined by Student’s *t*-test.

Aggregation and hyperphosphorylation of tau crucially contribute to AD pathogenesis, promoting the formation of NFTs and causing neuronal cell loss, resulting in cognitive deficits in AD patients. Thus, we measured total (t-) and phosphorylated (p-) tau levels in the cortex of mice using Western blotting. We found that the levels of t-tau and p-tau at multiple sites (S404, S422, and T231) in Tg2576/UCP1^−/−^ mice were significantly higher in Tg2576/UCP1^−/−^ mice than in Tg2576/ UCP1^+/+^ mice (Figure 4A). These findings indicate that the elevated body temperature observed in Tg2576/UCP1^−/−^ mice may enhance tau stability, ultimately leading to an increase in p-tau levels. Next, we investigated the molecular mechanisms by which the elevated body temperature in Tg2576/UCP1^−/−^ mice causes tau stability. Two major molecular chaperones, HSP70 and HSP90, can affect tau self-aggregation, clearance, and misfolding, leading to tau stability [27,28,29]. We observed higher HSP90 and HSP70 protein levels in Tg2576/UCP1^−/−^ mice than in Tg2576/ UCP1^+/+^ mice (Figure 4B). In contrast, protein levels of HSP60 were not significantly different between the two groups (Figure 4B). These findings suggest that the elevated body temperature observed in Tg2576/UCP1^−/−^ mice increases HSP70 and HSP90 protein levels, which may consequently stabilize the tau protein, leading to an increase in tau phosphorylation. Glycogen synthase kinase-3β (GSK3β), c-Jun N-terminal kinase (JNK), extracellular signal-regulated kinase (ERK), and p38 are known to enhance tau phosphorylation [30]. Furthermore, activation of these kinases is induced by heat shock stress responses [31]. Thus, we also measured the levels of total and phosphorylated kinases in the cortex of mice using Western blotting, and observed that Tg2576/UCP1^−/−^ mice showed an increase in the total levels of GSK3β, JNK, ERK, and p38 compared with those in Tg2576/UCP1^+/+^ mice, which paralleled the enhanced phosphorylation levels of these proteins (Figure 5). These findings indicate that high body temperature increases HSP70 and HSP90 levels, which in turn stabilizes tau and tau kinases, which ultimately leads to the hyperphosphorylation of tau.

### 2.5. Effect of UCP1 Deletion in Tg2576 Mice on Glial Cell Activation and Synaptic Protein Levels

**Figure 6:**
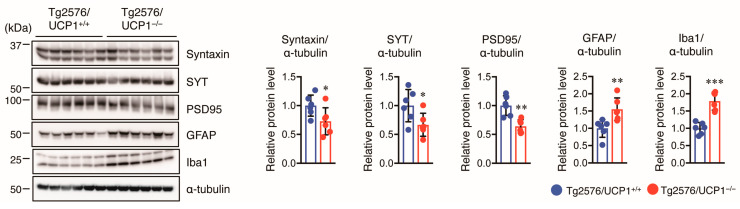
UCP1 deletion in Tg2576 mice enhances glial activation and decreases synaptic protein levels. Representative immunoblots showing the cortical levels of of syntaxin, SYT, PSD95, GFAP, Iba1, and α-tubulin in 17-month-old Tg2576/UCP1^+/+^ and Tg2576/UCP1^−/−^ mice (left panel). Relative protein levels quantified by densitometry (right panels). The results shown are mean ± SD, *n* = 6 for each group, * *p* < 0.05, ** *p* < 0.01, *** *p* < 0.001 vs. Tg2576/UCP1^+/+^, as determined by Student’s *t*-test.

Next, we investigated whether high body temperature in Tg2576/UCP1^−/−^ mice affected glial activation in the cortex of mice. We assessed GFAP (an astrocytic marker) and Iba1 (a microglial marker) protein levels using Western blotting and observed that both GFAP and Iba1 levels were higher in the cortex of Tg2576/UCP1^−/−^ mice than in Tg2576/UCP1^+/+^ mice (Figure 6). Furthermore, we assessed the effect of high body temperature on synaptic protein levels, such as syntaxin, synaptotagmin (SYT, presynaptic protein), and PSD95 (postsynaptic protein) in the cortex of mice using Western blot analysis. We found that these two protein levels in the cortex were significantly lower in Tg2576/UCP1^−/−^ mice than in Tg2576/UCP1^+/+^ mice (Figure 6). These findings indicate that the high body temperature observed in Tg2576/UCP1^−/−^ mice induces the activation of glial cells and decreases synaptic density.

## 3. Discussion

Several studies have shown that some patients with AD display increased nocturnal activity and body temperature [15,16,17,18,19]. However, the effect of hyperthermia on AD pathologies, such as Aβ deposition, tau phosphorylation, and neuroinflammation, is unclear. Several in vivo and in vitro studies have demonstrated that elevated body temperature could exacerbate AD pathology and disease progress. Validation of this hypothesis was difficult primarily due to a lack of experimental models. Recently, we demonstrated that Tg2576 mice housed at 30 °C for 13 months exhibited an increase in body temperature, which caused accelerated tau hyperphosphorylation and enhanced Aβ generation and deposition in the brain, leading ultimately to memory impairment, compared with those housed at 23 °C [26]. However, we could not exclude the possible effects of environmental factors associated with differences in ambient temperature (23 °C vs. 30 °C) in our previous study. UCP1^−/−^ mouse is a unique animal model to study the mechanism of body temperature regulation [21,24]. This mutant mouse shows a cold sensitive phenotype, but also has elevated body temperature [23], which is distinct from pathological hyperthermia such as fever. Therefore, in this study, we combined UCP1^−/−^ and Tg2576 mice to generate AD mice with a high body temperature, allowing us to study the effect of sustained elevated body temperature on AD-like pathologies at normal RT, and not at 30 °C. The mechanism underlying this phenotype in UCP1^−/−^ mice remains unclear, but the induction of UCP1-independent thermogenesis [32] and/or strong suppression of heat loss via vasoconstriction [24] may result in the increase in body temperature in UCP1^−/−^ mice. Although the rise in body temperature in Tg2576/UCP1^−/−^ mice was small, unlike that in high fever, the present model was useful for exploring the effects of slight but sustained increase in body temperature on AD-like pathologies. We found that, compared with Tg2576/UCP1^+/+^ mice, Tg2576/UCP1^−/−^ mice housed at 23 °C had exacerbated AD-like pathologies due to elevated levels of Aβ, HSPs, both phosphorylated and total tau, and tau kinases, as well as enhanced glial activation and decreased synaptic protein levels.

Enhanced Aβ generation via APP cleavage by β- and γ-secretase, leading to its aggregation, is the most pronounced hallmark of AD. BACE1 is a key enzyme in Aβ generation, in late-onset sporadic AD patients, and its activity and expression level are elevated in their brains [33]. In this study, we found that the high body temperature observed in Tg2576/UCP1^−/−^ mice increased soluble Aβ40 and Aβ42 and insoluble Aβ42 levels, and also increased BACE1 levels accompanied by increased CTFβ and sAPPβ levels. Our previous findings are similar to these findings in which a sustained increase in body temperature in Tg2576 mice housed at 30 °C significantly increased Aβ levels in the brain compared with mice housed at 23 °C, which was accompanied by BACE1 upregulation as well as increased CTFβ and sAPPβ levels. Although the link between increased BACE1 levels and high body temperature is not fully understood, the increased BACE1 levels may be linked with the JNK activation that is observed in the brains of Tg2576/UCP1^−/−^ mice. It has been reported that JNK activation contributes to the upregulation of BACE1, ultimately leading to Aβ generation [34]. Indeed, we found that the high body temperature observed in Tg2576/UCP1^−/−^ mice increased phosphorylated JNK levels, which may be a consequence of a high body temperature-induced oxidative stress response. Taken together, these results demonstrate that a high body temperature increases Aβ levels through a JNK-activated increase in BACE1 levels. We also found that high body temperature increased insoluble Aβ42, but not insoluble Aβ40, which may be caused by inhibition of Aβ degradation. The amyloid hypothesis suggests that the imbalance between Aβ clearance and production, or a decrease in proteolytic Aβ degradation causes an increase in Aβ levels [35]. Aβ can undergo proteolytic degradation via Aβ-degrading enzymes such as IDE and NEP. Furthermore, it has been reported that NEP degrades Aβ42 rather than Aβ40, so inhibition in Aβ42 degradation caused by the reduction in NEP levels [36], and the reduction in its activity and expression seen in the brain of AD inhibits Aβ42 degradation [37]. Thus, the increased Aβ42 levels could be attributed to the downregulation of NEP levels that impaired Aβ42 degradation.

Tau, a microtubule-associated protein, binds to stabilized microtubules and tau phosphorylation regulates tau binding to microtubules [38]. Consistent with our previous findings that a sustained increase in body temperature in Tg2576 mice housed at 30 °C showed increased total tau levels, resulting in enhanced tau phosphorylation, this study also obtained similar results; cortical levels of total and phosphorylated tau in Tg2576/UCP1^−/−^ mice were higher than those in Tg2576/UCP1^+/+^ mice. HSP70 and HSP90s are involved in tau stability by inhibiting tau degradation, which leads to hyperphosphorylation of tau [28,39] and the inhibitors of HSP70 and HSP90 attenuate tauopathy and degrade abnormal tau [40,41]. In this study, the result shows that cortical levels of HSP70 and HSP90 in Tg2576/UCP1^−/−^ mice were higher than those in Tg2576/UCP1^+/+^ mice, suggesting that high body temperature increased HSP levels, which may contribute to tau protein stabilization. It is known that tau kinases such as JNK, p38, ERK, and GSK3β regulate tau hyperphosphorylation [30]. In this study, we found that the activation of JNK, p38, ERK, and GSK3β was enhanced in the cortex of Tg2576/UCP1^−/−^ mice compared to that in Tg2576/UCP1^+/+^ mice, which is consistent with our previous findings. Therefore, high body temperature-induced kinase activation may enhance tau hyperphosphorylation.

Activated glial cells, including microglial cells and astrocytes, are considered the pathological hallmarks of AD. Neuroinflammation, a common feature of familial and sporadic AD, is caused by glial cell activation and triggers neurodegenerative disease progression, including AD [42,43]. In the brains of patients with AD and AD-like mouse models, reactive glial cells are present around Aβ plaques and play a vital role in the disease progression as they induce both Aβ deposition and the release of proinflammatory cytokines such as tumor necrosis factor (TNF-α), interleukin-6 (IL-6), and IL-1β [44]. Additionally, Aβ accumulation activates both astrocytes and microglia [45]. To determine whether higher body temperatures could alter glial activation, we evaluated the protein levels of Iba1 and GFAP, and observed that high body temperature in Tg2576/UCP1^−/−^ mice increased GFAP and Iba1 levels. Because of the limited brain samples, we were unable to evaluate the mRNA levels of proinflammatory cytokines; thus, further study is required to evaluate their levels.

Several lines of evidence have demonstrated that synaptic dysfunction is observed in various neurodegenerative diseases, including AD, and is the primary cause of AD [46,47]. Moreover, AD brains display a reduction of synaptic proteins by 20–40% [48,49]. Indeed, aggregated soluble Aβ can bind to postsynaptic and presynaptic proteins, impairing their function and leading to synaptic dysfunction [50]. In addition, synaptic protein loss has been found in neurofibrillary tangle-bearing neurons [51], suggesting that tau pathology is involved in synaptic protein loss. In this study, the result shows that high body temperature reduced the protein levels of presynaptic (SYT and syntaxin) and postsynaptic (PSD95) proteins, suggesting that increased body temperature reduces presynaptic and postsynaptic protein levels through an elevation in Aβ levels and tau phosphorylation. 

In conclusion, we validated a hypothesis that sustained high body temperature exacerbates AD-like pathology by using unique model UCP1^−/−^ mice, which have an elevated body temperature at normal RT in adult age. We found that compared with Tg2576/UCP1^+/+^ mice, Tg2576/UCP1^−/−^ mice housed at 23 °C had exacerbated AD-like pathologies due to increased Aβ, heat shock proteins (HSPs), total tau, phosphorylated tau, and phosphorylated tau kinases, as well as enhanced glial activation and decreased synaptic protein levels. Taken together, the current findings support those of our previous study and highlight that high body temperature is a risk factor for exacerbating AD-like pathologies.

## 4. Materials and Methods

### 4.1. Experimental Animals 

Female Tg2576 mice were purchased from TACONIC (Hudson, NY, USA), and the mice express humanized APP mutations (Swedish mutations K670N/M671L). These mice have high levels of Aβ plaque deposition around 7–9 months of age [52], and they show cognitive impairment at 6-month-old age [53]. UCP1-deficient (UCP1^−/−^) mice with a C57BL/6J genetic background have been previously described [23]. Tg2576 and UCP1^−/−^ mice were crossed to generate heterozygous Tg2576/UCP1^+/−^ breeders. These breeder mice were mated to obtain Tg2576/UCP+/+ and Tg2576/UCP1^−/−^ mice. All mice were housed under our animal laboratory conditions, 23 °C in a 12-h light/dark cycle, with free access to food and water ad libitum. All the experiments were performed in accordance with the National Institute of Health Guide for the Care and Use of Laboratory Animals and were carried out according to the Declaration of Helsinki Guiding Principles and were approved by Use of Laboratory Animals and the Animal Experimentation Committee of Chubu University (approval numbers: #1910037, #2010014, #2110009, #2210037, and #2310028) and the Committee of Nagoya City University Institutional Care (approval number: H29M-022H05).

### 4.2. Biotelemetry 

The core body temperature and physical activity of mice (Tg2576/UCP1^+/+^: one male and three females, Tg2576/UCP1^−/−^: three males and two females) were continuously recorded using a VitalView data acquisition system (Mini Mitter Co., Sunriver, OR, USA) as described previously [26]. In brief, mice were intra-abdominally implanted with a temperature-sensitive transmitter (G2 E-Mitter, Mini Mitter Co.) under general anesthesia, followed by the mice being allowed to recover for more than a week. The signals emitted by the transmitter (ER-4000, Mini Mitter Co.) were received by the VitalView software and converted to temperature and activity. Core body temperature and physical activity were monitored every minute and at a rate of 1–10 measurements per hour for 72 h, respectively.

### 4.3. Aβ ELISA

Aβ levels were measured as described in a previous report [26]. Briefly, cortical tissues of the left hemisphere (Tg2576/UCP1^+/+^: six females, Tg2576/UCP1^−/−^: two males and four females) were frozen in liquid nitrogen and stored at −80 °C until Aβ ELISA analysis. The cortical tissues were homogenized in Tris-buffered saline (TBS) containing a phosphatase inhibitor cocktail and a protease inhibitor cocktail (Wako Pure Chemical Industries, Osaka, Japan). The homogenates were centrifuged at 100,000 rpm for 20 min at 4 °C. The resulting supernatants were used to determine the soluble Aβ levels. For insoluble Aβ determination, the pellets were dissolved in 6 M guanidine chloride, following sonication, incubation for 1 h at RT, and centrifugation at 100,000 rpm for 20 min at 4 °C. Human/Rat β Amyloid (Aβ40) ELISA kits (#294-64071, FUJIFILM Wako Pure Chemical Corporation, Osaka, Japan) and Human/Rat β Amyloid (Aβ42) ELISA kits (#290-62601, FUJIFILM Wako Pure Chemical Corporation) were used according to the manufacturer’s instructions. The obtained Aβ levels were normalized to the brain tissue weight. 

### 4.4. Western Blot Analysis

The cortical tissues of the remaining hemibrains (n = 6 per group) were lysed in RIPA buffer (50 mM Tris-HCl, 150 mM NaCl, 1% Nonidet P-40, 0.5% sodium deoxycholate, and 0.1% SDS; pH 7.6) supplemented with protease and phosphatase inhibitor cocktails. The lysates were centrifuged at 12,000 rpm for 30 min, and protein concentration was analyzed using a BCA Protein Assay kit (Thermo Fisher Scientific, Rockford, IL, USA). Equal amounts of protein were separated using (SDS-PAGE) and separated proteins were transferred onto PVDF membranes (Millipore, Billerica, MA, USA). After blocking the membranes, the membranes were probed with the primary antibodies overnight at 4 °C, incubated with secondary antibodies. Immunoreactive bands were visualized using ImmunoStar Zeta or ImmunoStar LD (FUJIFILM Wako Pure Chemical Corporation) and imaged using Amersham Imager 680 (GE Healthcare Life Sciences, Marlborough, MA, USA). Alpha-tubulin was used as an internal control, and band intensities were quantified using the ImageJ software (NIH, Bethesda, Maryland, MA, USA). The primary and secondary antibodies used are shown in Supplementary Table S1.

### 4.5. Statistical Analysis

Statistical analyses were performed using GraphPad Prism9 software (GraphPad Software, San Diego, CA, USA). Results were analyzed using a two-tailed unpaired Student’s *t*-test for Aβ ELISA and Western blot analysis and repeated measures ANOVA with Fisher’s PLSD test for the measurement of core body temperature and physical activity. Data are expressed as the mean ± SD. Significance was defined at *p* < 0.05.

## Data Availability

All data used in this study are available from the corresponding authors on reasonable request.

## Supplementary Materials

The supporting information can be downloaded at: https://www.mdpi.com/article/10.3390/ijms24032741/s1.
