# Supplementary material for: Deletion of UCP1 in Tg2576 Mice Increases Body Temperature and Exacerbates Alzheimer’s Disease-Related Pathologies
Source: Int J Mol Sci. 2023 Feb 1;24(3):2741. doi: 10.3390/ijms24032741 (PMC9917061; doi:10.3390/ijms24032741)
Supplement: Supplementary file 1 [file ijms-24-02741-s001.zip › ijms-2145728-supplementary.pdf]

Supplementary Table S1. List of antibodies used in the study.

| Antibodies | Species | Dilution | Source | Cat. No. |
|---|---|---|---|---|
| Primary antibodies | | | | |
| ABCA1 | Mouse | 1:1000 | Abcam | AB18180 |
| ADAM10 | Rabbit | 1:1000 | Millipore | AB19026 |
| ApoE | Goat | 1:1000 | Millipore | AB947 |
| APP | Mouse | 1:1000 | Millipore | MAB348 |
| BACE1 | Mouse | 1:1000 | R&D | MAB931 |
| APP-CTF | Rabbit | 1:1000 | Sigma | A8717 |
| IDE | Rabbit | 1:1000 | Covance | PRB-282C |
| NEP | Goat | 1:1000 | R&D | AF1126 |
| PS1 | Mouse | 1:1000 | Millipore | MAB5232 |
| sAPPβ | Mouse | 1:1000 | IBL | 10321 |
| total tau | Mouse | 1:1000 | BioLegend | 806401 |
| Tau p-S404 | Rabbit | 1:1000 | Invitrogen | 44758ZG |
| Tau p-S422 | Rabbit | 1:1000 | Invitrogen | 44764ZG |
| Tau p-T231 | Mouse | 1:1000 | Invitrogen | MN1040 |
| HSP60 | Mouse | 1:2000 | BD Biosciences | 611562 |
| HSP70 | Rabbit | 1:2000 | Bioworld | BS2741 |
| HSP90 | Mouse | 1:2000 | BD Biosciences | 610418 |
| Total GSK3α/β | Rabbit | 1:1000 | Cell Signaling | 5676 |
| p-GSK3α/β (S21/9) | Rabbit | 1:2000 | Cell Signaling | 8566 |
| Total JNK | Rabbit | 1:1000 | Cell Signaling | 9258 |
| p-JNK (T183/Y185) | Mouse | 1:1000 | Cell Signaling | 9255 |
| Total ERK | Rabbit | 1:2000 | Cell Signaling | 9102 |
| p-ERK (T202/Y204) | Mouse | 1:1000 | Cell Signaling | 9106 |
| Total p38 | Rabbit | 1:1000 | Cell Signaling | 9212 |
| p-p38 (T180/Y182) | Rabbit | 1:1000 | Cell Signaling | 9211 |
| α-Tubulin | Rabbit | 1:2000 | Cell Signaling | 2148 |
| Syntaxin | Mouse | 1:1000 | Sigma | SAB4200841 |
| Synaptotagmin (SYT) | Mouse | 1:1000 | BD Biosciences | 610433 |
| PSD95 | Rabbit | 1:1000 | Cell Signaling | 3450 |
| GFAP | Mouse | 1:1000 | Sigma | G3893 |
| Iba1 | Rabbit | 1:1000 | Wako | 019-19741 |
| Secondary antibodies | | | | |
| Anti-rabbit IgG, HRP-linked | Goat | 1:5000 | Cell Signaling | 7074 |
| Anti-mouse IgG, HRP-linked | Goat | 1:5000 | Cell Signaling | 7076 |
